# Experiments with a View of Ascertaining the Effect of Tying the Ductus Communis Choledochus

**Published:** 1826-10

**Authors:** Herbert Mayo


### ON THE USE OF THE BILE.

*Experiments with a view of ascertaining the Effect of Tying the Ductus Communis Choledochus.* By Herbert Mayo, Esq. &c.

It must be well known to your readers, that Mr. Brodie found the production of chyle prevented when the ductus communis choledochus was tied. The experiment was performed upon young cats: on its repetition, the very remarkable circumstance was noticed, that, if the animal survived a sufficient length of time, the duct was restored by a process similar to that which Mr. Travers had observed upon tying a ligature round a portion of intestine. The attention evidently bestowed upon this subject by Mr. Brodie, which its importance well deserves, appeared to preclude the possibility of error in his researches. It was not, therefore, without considerable surprise that I read a note in the last edition of M. Magendie's original and excellent Treatise on Physiology, of a part of which the following is a translation:

"I have repeated the experiment of tying the ductus communis choledochus in adult animals: the greater number died of the consequences of the wound of the abdomen, and the violence attending the ligature of the duct. But in two cases, in which the animals survived several days, I was enabled to satisfy myself that white chyle had been formed, and fæcal matter produced. The fæces had not the usual colour; which is not surprising, as they contained no bile. The animals had not become jaundiced."

The preceding statement seemed to throw the question, which Mr. Brodie's experiments had for a time decided, into its former uncertainty; and, in order to advance a step nearer truth, nothing appeared left but to go over the same ground a third time. Accordingly, with the assistance of my friend Mr. Cæsar Hawkins, I repeated the experiment, and met with the following results:

The ductus communis choledochus was tied in three cats, each about four months old, which had fasted for twenty-four hours previously. They each took food after the operation, which they threw up; but they afterwards again took food, consisting of milk and raw or boiled meat, and continued to eat occasionally with a natural appetite.

One of these animals was killed between five and six hours after the duct had been tied. The stomach contained a full meal of meat, consisting in part of morsels, which were softened by the action of the gastric juice, but had undergone no further alteration; in part of a pulpy mass of a reddish-grey colour, in part of a brownish-grey viscid liquid, in which innumerable small globules of oil floated. The small intestines were perfectly empty.

The second died within fifty hours after the experiment. The stomach contained a small quantity of half-digested food; the small intestines contained scarcely a trace of a greyish semifluid substance, which here and there admitted of being scraped from the villous surface.

The third was killed three days after the operation. The stomach contained half-digested food; the small intestines contained a quantity of a greyish viscid liquid, very like the liquid contents of the stomach. The great intestines, in this and the preceding instance, were distended with a greyish, tenacious, and highly offensive semifluid matter.

An adult dog, in which the duct had been tied, was found dead on the second morning of the experiment. The mucous membrane of the stomach and bowels was inflamed; the stomach contained water only; the small intestines held a quantity of yellowish ropy liquid.

Finally, the duct was tied in two young dogs, which had fasted for twenty-four hours. One died, the second was killed, about forty-eight hours after the operation. Both had eaten boiled flesh, and had taken milk. In the first, the stomach contained half-digested food; and the small intestines contained a quantity of grey liquid, separate from a viscid ropy material, that adhered to the villous surface. In the second, the stomach contained a frothy mucus only; but the small intestine was moderately distended with a quantity of yellowish liquid.

The animals which were killed were immediately examined: those which died were examined from four to five hours afterwards. In each case the duct was found to have been accurately secured: the gall-bladder and gall-ducts were distended with bile; and there was no trace whatever of chyle in the lacteal vessels.

The general coincidence of these results with those obtained by Mr. Brodie, leads me to suppose that M. Magendie may possibly have overlooked some source of error in his experiments. In one of the experiments which I have narrated, the animal, when pithed, bled profusely; and, when the mesentery was first examined, the empty arteries upon it presented exactly the appearance of lacteals. Possibly M. Magendie was deceived by a similar circumstance; or it might have happened that the duct had been restored in the two instances he mentions, and that the bile had thus again found its way into the duodenum.

In making these remarks, I am endeavouring only to reconcile conflicting accounts, where the good faith of the narrators is beyond all doubt, and where the scrupulous accuracy of both parties is so well acknowledged as to render any additional testimony futile, except in the present curious instance, where their observations happen to be at variance. Let M B. try their experiments on each other.

